# Eine seltene Ursache für das akut blockierte Kniegelenk: die superiore Patellaluxation

**DOI:** 10.1007/s00113-020-00907-2

**Published:** 2020-10-16

**Authors:** Johannes Glasbrenner, Thorben Briese, Michael J. Raschke, Elmar Herbst, Christoph Kittl

**Affiliations:** Klinik für Unfall‑, Hand- und Wiederherstellungschirurgie, Universitätsklinikum Münster, Albert-Schweitzer-Campus 1, 48147 Münster, Deutschland

**Keywords:** Superiore Patellaluxation, Kniegelenksblockade, Flexionsdefizit, Geschlossene Reposition, Osteophyten, Superior dislocation of the patella, Locking of the knee joint, Flexion deficit, Closed reduction, Osteophyts

## Abstract

Es wird über den Fall eines 56-jährigen Patienten berichtet, der sich ohne Traumaanamnese mit akuter Blockade des Kniegelenks in Streckung vorstellte. Anamnese, klinische Untersuchung und Röntgen führten zur Diagnose einer superioren Patellaluxation. Nach geschlossener Reposition konnte bei beschwerdefreiem Patienten eine Entlassung ohne Hilfsmittel erfolgen. Die Kenntnis dieser seltenen Ursache des akut blockierten Kniegelenks kann helfen, aufwändigere apparative Untersuchungen einzusparen und eine zügige Behandlung und Beschwerdelinderung herbeizuführen.

## Abstract (en)

The case of a 56-year-old male patient with locking of the knee in extension without trauma is presented. Patient history, clinical examination and radiographs confirmed a superior dislocation of the patella. Closed reduction led to full weight bearing with free range of motion without further complaints. Knowledge of this uncommon cause of a locked knee joint leads to immediate treatment avoiding complex imaging modalities, such as computed tomography (CT) or magnetic resonance imaging (MRI). No further treatment was necessary in the majority of the cases published in the literature.

## Anamnese

Es wird über den Fall eines 56-jährigen Patienten berichtet. Dieser hatte am Heiligabend beim Ausräumen der Spülmaschine eine maximale Streckung des Kniegelenks durchgeführt, wodurch es ohne weitere Krafteinwirkung zu einer Blockade in Streckung gekommen war. Er stellte sich unmittelbar in Begleitung seiner Tochter in der unfallchirurgischen Notfallambulanz vor. Vorerkrankungen und Medikamenteneinnahmen wurden verneint. Verletzungen der Kniegelenke seien bisher nicht aufgetretenen und Operationen an den unteren Extremitäten nicht durchgeführt worden. Weder auf der betroffenen noch auf der kontralateralen Seite seien bis zum heutigen Tage Beschwerden, Blockaden oder eine Patellaluxation aufgetreten.

## Befund und Diagnose

**Figure 1:**
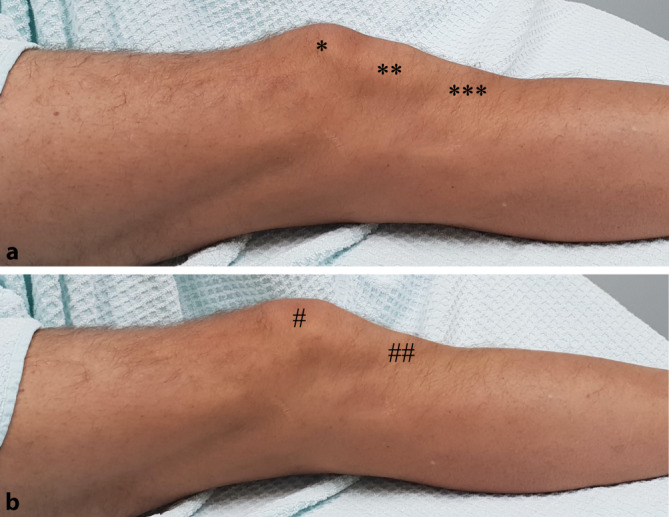

Bereits beim Betreten der Ambulanz fiel die Blockade des Kniegelenks in Streckung auf. Die axiale Belastung des betroffenen Beins war beschwerdefrei möglich. Bei der Inspektion imponierte eine Anhebung des proximalen Patellapols (Abb. 1). Ein knöcherner Druckschmerz war nicht auszulösen; eine Schwellung oder ein Gelenkerguss fand sich nicht. Die Palpation ergab einen prominenten proximalen und einen versunkenen distalen Patellapol sowie eine Blockade des Kniegelenks in Streckstellung mit hartem Anschlag beim Versuch der Beugung. Die periphere Durchblutung, Motorik und Sensibilität zeigten sich intakt.

**Figure 2:**
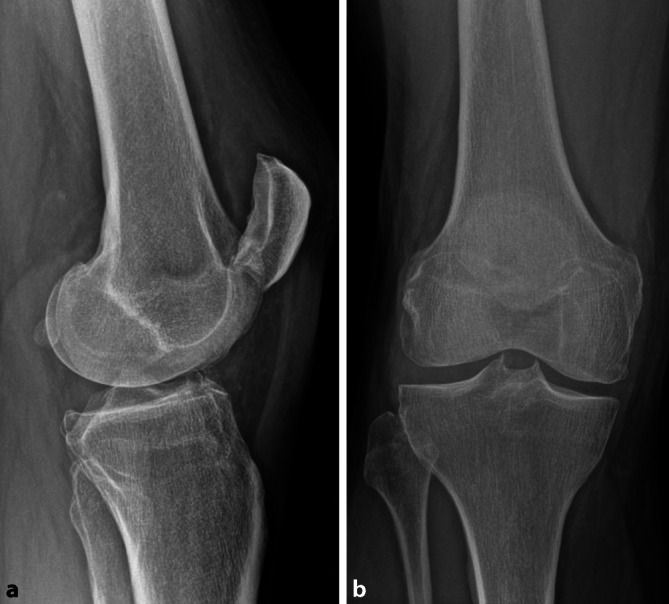

Das Röntgen des Kniegelenks in 2 Ebenen zeigte eine um die Transversalachse verkippte Patella. Im lateralen Strahlengang war zu erkennen, dass die Patella im Sinne einer superioren Luxation mit einem osteophytären Anbau am distalen Ende der patellaren Gelenkfläche am proximalen Ende der Trochlea femoris zentral eingehakt war (Abb. 2).

## Therapie und Verlauf

Nach Aufklärung des Patienten über das Krankheitsbild und das weitere Vorgehen erfolgte die Reposition unter i.v.-Analgesie in Rückenlage mit Lagerung des Kniegelenks in maximal möglicher Extension. Bei entspanntem Streckapparat wurde die Patella unter dosiertem Druck von anterior auf den distalen Patellapol nach proximal geschoben und so aus der Einklemmung befreit.

**Figure 3:**
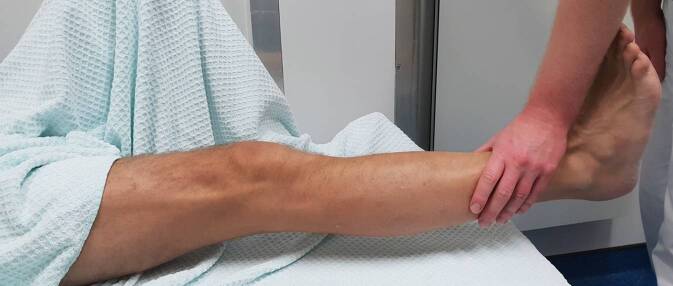

Dies gelang ohne Komplikationen; unmittelbar war eine beschwerdefreie aktive Mobilisation mit freier Beugung und Streckung des Kniegelenks möglich (Abb. 3).

In der folgenden funktionellen Untersuchung zeigte sich ein ligamentär stabiles Gelenk. Der Apprehensionstest war negativ, und die Patella richtete sich bei negativem „J-sign“ regelhaft im femoralen Gleitlager aus. Hinweise auf eine Verletzung des medialen oder lateralen Retinakulums ergaben sich nicht. Eine Krepitation konnte nicht provoziert werden. Auch nach der Reposition zeigten sich die periphere Durchblutung, Motorik und Sensibilität intakt.

Der Patient wurde über die Notwendigkeit einer weiterführenden Bildgebung (Computertomographie [CT] oder Magnetresonanztomographie [MRT]) bei rezidivierenden Beschwerden aufgeklärt. Mittelfristig würde bei wiederkehrenden Luxationen eine arthroskopische Resektion der osteophytären Anbauten abgewogen werden. An diesem Abend konnte der Patient beschwerdefrei ohne Hilfsmittel entlassen werden. Erneute Beschwerden oder eine Rezidivluxation sind bis zum Zeitpunkt 20 Monate nach superiorer Patellaluxation nicht eingetreten.

## Diskussion

Im vorgestellten Fall konnte bei akuter Blockade des Kniegelenks nach Anamnese, klinischer Untersuchung und Röntgendiagnostik die Diagnose einer superioren Patellaluxation gestellt werden. Unter i.v.-Analgesie gelang eine komplikationslose geschlossene Reposition. Die Nachbehandlung erfolgte unter beschwerdeadaptierter Vollbelastung. Eine erneute Blockade des Kniegelenks oder anderweitige Beschwerden ist/sind bis 20 Monate postoperativ nicht aufgetreten.

Während die laterale Patellaluxation eine relativ häufige Entität darstellt, sind bisher lediglich 29 Fälle einer superioren Patellaluxation beschrieben [1–11, 13–27, 29–31]. In der Literatur variiert das Alter der Patienten zwischen 19 und 88 Jahren, wobei das Geschlecht oder der BMI keine Risikofaktoren darzustellen scheinen. Als ursächlicher Mechanismus wird in der Mehrzahl der Fälle eine reine Hyperextension beschrieben. Seltener lag zusätzlich ein Anpralltrauma der Patella vor [17, 22]. Eine fortgeschrittene Degeneration und eine Patella alta werden als Risikofaktoren für das Eintreten einer superioren Luxation beschrieben [6, 17]. Cusco et al. berichten von einem Fall nach hoher tibialer Umstellungsosteotomie mit konsekutiver Veränderung der Patella-Höhe und des Q‑Winkels [5]. Entsprechend dem hier präsentierten Fall handelt es sich jedoch in der Mehrzahl der in der Literatur beschriebenen Fälle um die erste, klinisch relevante Manifestation degenerativer Veränderungen im Patellofemoralgelenk.

Die Verkippung der Patella mit Abhebung des proximalen Pols vom distalen Femur wird als pathognomonisch angesehen und kann bei schlankem Weichteilmantel bereits bei der Inspektion erkannt werden (Abb. 1; [29]). Eine Schwellung oder ein relevanter Gelenkerguss liegt in der Regel nicht vor. Eine funktionelle Untersuchung des Gelenks und der Ligamente ist aufgrund der Blockade des Kniegelenks nur sehr eingeschränkt möglich.

In allen beschriebenen Fällen wurde ein Röntgen des Kniegelenks in 2 Ebenen durchgeführt, wobei insbesondere im lateralen Strahlengang die Verkippung der Patella evident wurde (Abb. 2). Gakhar et al. untermauerten den Befund zusätzlich mit einer Computertomographie des Kniegelenks [9]. Iorwerth et al. entschieden sich zur differenzialdiagnostischen Abgrenzung einer Verletzung des Streckapparats für ein MRT vor Reposition [13].

Aus Sicht der Autoren und im Einklang mit den beschriebenen Fällen kann nach Anamnese, klinischer Untersuchung und Röntgen auf weitere Diagnostik zunächst verzichtet und unmittelbar die geschlossene Reposition eingeleitet werden.

Zur geschlossenen Reposition werden in der Literatur neben der i.v.-Analgesie eine i.m.- oder i.a.-Applikation beschrieben [7, 14]. Yip et al. schlagen ein Analgesiestufenschema zur Reposition vor [31]: Sollte die i.v.-Analgesie nicht ausreichen, kann eine Lokalanästhesie oder im Weiteren eine Vollnarkose abgewogen werden. Nur in einem beschriebenen Fall war primär ein chirurgisches Vorgehen mit offener Resektion der osteophytären Anbauten zur Reposition notwendig [20]. In den weiteren 28 Fällen konnte, entsprechend dem hier präsentierten Fall, eine geschlossene Reposition durchgeführt werden.

Die Autoren favorisieren eine opioidbasierte i.v.-Analgesie mit Überwachung der Vitalparameter. Die Lagerung erfolgt in Rückenlage mit Hyperextension des Kniegelenks, ggf. durch Anheben der Ferse, und Entspannung der Muskulatur. Der Behandler greift den distalen Patellapol mit beiden Daumen von inferior und übt Druck nach posterior und superior aus, um den eingehakten distalen Patellapol unter dem Repositionshindernis zu befreien.

Nach erfolgreicher Reposition sollten erneut eine dezidierte funktionelle Untersuchung des Kniegelenks sowie eine Überprüfung der peripheren Durchblutung, Motorik und Sensibilität durchgeführt werden. Da Verletzungen der Patellarsehne sowie der Quadrizepssehne in Betracht gezogen werden müssen, sollte besonderes Augenmerk auf die funktionelle Untersuchung des Streckapparats gelegt werden (Abb. 3). Die Sonographie kann hierbei helfen, die Integrität der Quadrizeps- und der Patellarsehne einzuschätzen [3]. Bei klinischem Verdacht auf eine osteophytäre Fraktur durch das Repositionsmanöver sollte ein erneutes Röntgen des Kniegelenks erwogen werden [10]. Takai et al. führten nach der Reposition eine zusätzliche fluoroskopische Untersuchung durch, um das Risiko für eine Rezidivluxation einzuschätzen [26].

Differenzialdiagnostisch ist die Abgrenzung zur lateralen Patellaluxation hinsichtlich der notwendigen Diagnostik und Therapie von großer Bedeutung [6, 12, 19, 28]. Bei Vorliegen einer lateralen Patellaluxation sollte gemäß aktueller AWMF-Leitlinie aufgrund des relevanten Risikos osteochondraler Begleitverletzungen und, um das Ausmaß der Schädigung des medialen Halteapparats und mögliche Gelenkbinnenschäden einzuschätzen, in jedem Fall ein MRT des Kniegelenks durchgeführt werden (https://www.awmf.org/leitlinien/detail/ll/012-024.html). Nach einer differenzierten Analyse der Risikofaktoren für eine Rezidivluxation kann im Fall einer lateralen Patellaluxation auch unabhängig von den subjektiven Beschwerden des Patienten eine operative Therapie empfohlen werden [12, 28].

Im Gegensatz zur lateralen Luxation verlässt die Patella bei der superioren Luxation ihren nativen Pfad nicht, sondern verhakt sich in Extension vor dem Eintreten in die knöcherne Führung der Trochlea femoris mit dem distalen Pol unter dem kranialen Rand der Trochlea. Entsprechend scheint das Risiko für klinisch relevante oder therapiebedürftige Begleitverletzungen bei superiorer Patellaluxation wesentlich geringer zu sein als bei der lateralen Patellaluxation.

In der Literatur kam es in 25 der 28 Fälle, in denen eine geschlossene Reposition bei superiorer Luxation möglich war, unmittelbar zu einer beschwerdefreien Mobilisation. Eine Nachbehandlung mit Teilbelastung und Orthese [7] oder eine Einschränkung der Streckung durch eine 4‑Punkt-Hartrahmenorthese [31] wurde im Einzelfall beschrieben. Eine Rückkehr in den Kontaktsport verlief im Fall eines Rugbyspielers 2 Monate nach geschlossener Reposition komplikationslos [22]. In 5 der 29 beschriebenen Fälle wurde im Verlauf bis ein Jahr nach superiorer Luxation eine arthroskopische Resektion osteophytärer Anbauten durchgeführt [5, 10, 26, 27, 29]. Auch mittelfristig werden in der Literatur überwiegend rezidivfreie günstige Verläufe bis zu 3 Jahre nach superiorer Patellaluxation beschrieben.

Vor dem Hintergrund der aktuellen Literatur – und entsprechend dem dargelegten Fall – sind das Vorgehen nach superiorer Luxation und die Notwendigkeit apparativer Diagnostik vom klinischen Befund und von den Beschwerden des Patienten abhängig. Nach komplikationsloser Reposition und blander klinischer Untersuchung kann post repositionem auf eine weiterführende apparative Diagnostik (Röntgen, CT, MRT) verzichtet und eine beschwerdeadaptierte Vollbelastung ohne Hilfsmittel eingeleitet werden.

Eine Schnittbildgebung ist im Fall eines Rezidivs oder bei neu auftretenden Beschwerden notwendig [27, 29]. Zur Planung einer (arthroskopischen) Resektion osteophytärer Anbauten ist dann ein CT des Kniegelenks hilfreich, das MRT hingegen bietet den Vorteil, begleitende Gelenkbinnenschäden aufzudecken.

In jedem Fall wird eine Aufklärung des Patienten über den Mechanismus der superioren Luxation als sinnvoll angesehen, um erneuten Blockaden zu vermeiden.

## Schlussfolgerung

Die superiore Luxation der Patella stellt eine seltene Ursache des akut blockierten Kniegelenks dar. Die Diagnosestellung erfolgt auf Basis von Anamnese, klinischer Untersuchung und Röntgen des Kniegelenks in 2 Ebenen. In der überwiegenden Mehrzahl der Fälle ist die Reposition ohne größeren Aufwand zügig durchführbar. Die Kenntnis des Krankheitsbildes und die differenzialdiagnostische Abgrenzung zur lateralen Patellaluxation können helfen, Untersuchungsmodalitäten wie CT oder MRT zunächst zu vermeiden und eine zügige Behandlung und Beschwerdelinderung herbeizuführen. Weiterführende Maßnahmen und die Vorstellung beim Spezialisten sind bei rezidivierenden Beschwerden notwendig.

## Fazit für die Praxis

- Die superiore Luxation der Patella stellt eine seltene Differenzialdiagnose des akut blockierten Kniegelenks dar.
- Eine Patella alta oder beginnende degenerative Veränderungen kann/können eine superiore Patellaluxation begünstigen.
- Die Diagnosestellung erfolgt auf Basis von Anamnese, klinischer Untersuchung und Röntgen des Kniegelenks in 2 Ebenen.
- Eine geschlossene Reposition ist in der überwiegenden Mehrzahl der Fälle, ggf. unter medikamentöser Analgesie, ohne größeren Aufwand zügig durchführbar.
- Die Differenzialdiagnostische Abgrenzung zur lateralen Patellaluxation ist entscheidend für das weitere Vorgehen.
- Bei anhaltenden Beschwerden oder Rezidivluxation sind weiterführende Maßnahmen wie eine Schnittbildgebung und die Vorstellung beim Spezialisten notwendig.
